# Editorial: Toxicity mechanisms, exposure, toxicokinetic and risk assessment aspects of metals, toxic for animals and humans, volume III

**DOI:** 10.3389/fphar.2025.1690145

**Published:** 2025-09-16

**Authors:** Yanzhu Zhu, Fatma Mohamady El Demerdash, Xu Yang, Michel Mansur Machado, Alex Boye, Xudong Sun

**Affiliations:** 1 College of Animal Science and Technology, Jilin Agricultural Science and Technology University, Jilin, China; 2 Institute of Graduate Studies and Research, Alexandria University, Alexandria, Egypt; 3 College of Veterinary Medicine, Henan Agricultural University, Zhengzhou, China; 4 Course of Pharmacy, Federal University of Pampa, Uruguaiana, Rio Grande do Su, Brazil; 5 Department of Medical Laboratory Science, School of Allied Health Sciences, College of Health and Allied Sciences, University of Cape Coast, Cape Coast, Ghana; 6 College of Animal Science and Technology, Heilongjiang Bayi Agricultural University, Daqing, China

**Keywords:** exposure, metals, toxicity mechanisms, toxicokinetic, risk assessment

Living organisms including humans and animals are unavoidably exposed to the chemical space (xenobiotics, e.g., chemicals in the air, water and the soil). Some xenobiotics such as food and drugs which are consciously consumed by humans and animals are useful in that they promote growth and development to impact health and wellbeing. However, other xenobiotics including but not limited to heavy metals (toxicants) upon exposure to humans and animals may cause various degrees of toxicity affecting quality of human life and also reducing the productive quality of farm animals used as food. Despite the known consequences of xenobiotic exposures, the current knowledge on xenobiotics, specifically heavy metals with regards to their diversity, toxicity mechanisms, and exposure-health risk relationships remain inadequate to inform regulatory and policy review necessary for managing environmental and occupational exposures. Therefore, this thematic topic was informed by the knowledge gap on metals and metal-induced toxicity.

Metals are ubiquitously distributed in the environment therefore humans and animals are unavoidably exposed. A study emphasized how arsenic exposure impairs a number of sensory systems in the body, therefore the need to consider arsenic exposure as a major public health concern that should warrant strict monitoring in the environment and work place. Corroboratively, urinary arsenic levels were linked to shifts in hearing threshold of adults in the United States (Long et al.). A study investigated environmental cadmium and the risk of kidney functional impairment and observed that cadmium exposure increased the risk of kidney stones (Ren et al.). Similarly, cadmium and polystyrene microplastics, two ubiquitous environmental contaminants synergistically upon co-exposure impair autophagic flux to increase lipid accumulation (Chen et al.) and these observations emphasize the need for minimizing cadmium exposures at the workplace as well as in other exposure routes. Dimethylacetamide (DMAC) find application in the dye, leather and paint industries as a solvent. Diaminodiphenyl ether (4, 4 – ODA), a precursor for synthesis of curing agents and dyes is a known inhibitor of the methemoglobin reduction system. A pipe leak from a plant containing DMAC and trace concentrations of 4, 4 – ODA was linked to serious clinical toxicity (respiratory failure, methemoglobinemia, hemolytic anemia, and liver injury) in two exposed people in 2024. Although the two exposed people were treated and discharged, later one was diagnosed with encephalopathy 16 days post-exposure while the other developed peripheral neuropathy 58 days post-exposure (Zhang et al.). Metal exposures do not discriminate and in this light farm animals were not left out. A study assessed heavy metal exposure in farm animals and how it impacts on the reproductive and productive performance of livestock. Interestingly, it was observed that heavy metal exposure to livestock negatively impacted on reproductive and productive performance of the livestock raising concern on the long-term health consequences for humans who consume animal products from such exposed livestocks (Afzal and Mahreen).

Treatment-related toxicity has been a public health concern, particularly use of some drugs in the management of human diseases. Xiong et al. studied the adverse effects associated with the use of sunitinib and ripretinib in the treatment of gastrointestinal stromal tumors analyzing data from the FDA adverse event reporting system (FAERS). The COVID-19 pandemic exposed the vulnerability of global emergency health preparedness and ever since efforts have been made to contain the COVID-19 pandemic. A key effort hovered around the avalanche of antiviral agents that were produced in record time to mitigate COVID-19. One of such antiviral agents was Azvudine. Hepatotoxicity was strongly associated with Azvudine (Xiong et al.). This observation may limit the clinical use of Azvudine particularly in COVID-19 patients with existing liver disease. It is instructive from this study to consider adding Azvudine to the Therapeutic Drug Monitoring (TDM) list in order to reduce toxicity whiles optimizing Azvudine use.

Mechanistic elucidation of metal poisoning is crucial to inform therapy. Guo et al. demonstrated mechanisms underlying cadmium and polysterene induced hepatotoxicity upon co-exposure. Carnitine palmitoyl transferase I (CPT1) was implicated in cadmium and polystyrene microplastics co-exposure–induced hepatotoxicity in C57BL/6 mice and AML12 cells. Impaired copper homeostasis was shown to trigger cuproptosis in heart failure by impairing mitochondrial function (Liu et al.). Monocrotaline (MCT), a major pyrrolizidine alkaloid, is a known hepatotoxin. MCT induced autophagy in hepatocytes by inhibiting PI3K/AKT/mTOR signaling pathway (Guo et al.). Cantharidin (CTD), derived from *Mylabris*, a Chinese traditional medicine demonstrated anti-tumor effects but caused hepatotoxicity mediated through DDIT4/mTOR signaling pathway (Tang et al.).

Exploring novel remedies for metal poisoning is crucial in the present context of widespread metal exposures due to industrialization and technological advancement. Sentinel selenium doses were used to improve cadmium and lead induced decrease in hand grip strength (Liang et al.). Similarly, naringenin ameliorated cadmium chloride-induced renal injury by modulating autophagy, oxidative stress and endoplasmic reticulum stress (Shi et al.).

Collectively, all the studies presented in this thematic topic suggest the urgent need for elaborate understanding of exposure dynamics of heavy metals, mechanistic insights into heavy metal exposure-toxicity relationships. Establishment of exposure route-specific and soft tissue-specific limits for these heavy metals may be crucial in reducing health risk associated with these ubiquitously distributed toxicants. Health policymakers and regulatory agencies in the environmental chemical control space need to review existing policies and regulations. For instance, mandatory regular monitoring of heavy metals in vulnerable human populations as well as in matrices such as air, water, food products, and soil in order to prevent long term health risk upon exposure.

