# Correction: Editorial: Toxicity mechanisms, exposure, toxicokinetic and risk assessment aspects of metals, toxic for animals and humans, volume III

**DOI:** 10.3389/fphar.2026.1734557

**Published:** 2026-03-02

**Authors:** Yanzhu Zhu, Fatma Mohamady El Demerdash, Xu Yang, Michel Mansur Machado, Alex Boye, Xudong Sun

**Affiliations:** 1 College of Animal Science and Technology, Jilin Agricultural Science and Technology University, Jilin, China; 2 Institute of Graduate Studies and Research, Alexandria University, Alexandria, Egypt; 3 College of Veterinary Medicine, Henan Agricultural University, Zhengzhou, China; 4 Course of Pharmacy, Federal University of Pampa, Uruguaiana, Rio Grande do Su, Brazil; 5 Department of Medical Laboratory Science, School of Allied Health Sciences, College of Health and Allied Sciences, University of Cape Coast, Cape Coast, Ghana; 6 College of Animal Science and Technology, Heilongjiang Bayi Agricultural University, Daqing, China

**Keywords:** exposure, metals, toxicity mechanisms, toxicokinetic, risk assessment

In the published article, there was a mistake in the author list.

Author Xinwei Li was erroneously included as an author in the published article and author Xudong Sun was erroneously omitted as an author.

Additionally, author Alex Boye was erroneously assigned as corresponding author. The correct corresponding author is Yanzhu Zhu.

Finally, the order of authors in the author list of the published paper was erroneously given as:

Alex Boye*, Yanzhu Zhu, Xinwei Li, Michel Mansur Machado, Fatma M. El Demerdash, Xu Yang.

The final correct author list reads:

Yanzhu Zhu, Fatma Mohamady El-Demerdash, Xu Yang, Michel Mansur Machado, Alex Boye, Xudong Sun.

The Author Contributions Statement has been corrected to read:

YZ: Conceptualization, Resources, Supervision, Writing – review and editing. FE-D: Conceptualization, Supervision, Writing – review and editing. XY: Supervision, Writing – review and editing. MM: Conceptualization, Supervision, Writing – review and editing. AB: Conceptualization, Writing – original draft, Writing – review and editing. XS: Resources, Supervision, Writing – review and editing.

In the published article, there was a mistake in the affiliations list.

Affiliation “College of Animal Science and Technology, Heilongjiang Bayi Agricultural University, Daqing, China” was omitted for author Xudong sun. This affiliation has now been added.

Affiliation “College of Animal Science and Technology, Jilin Agricultural Science and Technology University, Jilin, China” was omitted from the paper. This affiliation has now been added for author Yanzhu Zhu.

Affiliation “Jilin Agriculture Science and Technology College, Jilin City, Jilin,” was erroneously assigned to author Yanzhu Zhu. This affiliation has now been removed from the paper.

Affiliation 4 “Course of Pharmacy, Federal University of Pampa, Uruguaiana, Rio Grande do Sul, Brazil” was erroneously given as “Universidade Federal do Pampa, Uruguaiana, Brazil”.

Affiliation 2 “Institute of Graduate Studies and Research, Alexandria University, Alexandria, Egypt” was erroneously given as “College of Environmental Studies, Institute of Graduate Studies and Research, Alexandria University, Alexandria, Egypt”.

Affiliation 3 “College of Veterinary Medicine, Henan Agricultural University, Zhengzhou, China” was erroneously given as “Department of Veterinary Medicine, Henan Agricultural University, Zhengzhou, Henan, China”.

The original article has been updated.

